# Uncovering Research Trends of Phycobiliproteins Using Bibliometric Approach

**DOI:** 10.3390/plants10112358

**Published:** 2021-11-01

**Authors:** Hui Teng Tan, Fatimah Md. Yusoff, Yam Sim Khaw, Siti Aqlima Ahmad, Noor Azmi Shaharuddin

**Affiliations:** 1 Aquatic Animal Health and Therapeutics Laboratory, Institute of Bioscience, Universiti Putra Malaysia, Serdang 43400, Selangor, Malaysia; huiteng.tan28@gmail.com (H.T.T.); yskhaw@gmail.com (Y.S.K.); 2 International Institute of Aquaculture and Aquatic Sciences, Universiti Putra Malaysia, Port Dickson 71050, Negeri Sembilan, Malaysia; 3 Department of Aquaculture, Faculty of Agriculture, Universiti Putra Malaysia, Serdang 43400, Selangor, Malaysia; 4 Department of Biochemistry, Faculty of Biotechnology and Biomolecular Sciences, Universiti Putra Malaysia, Serdang 43400, Selangor, Malaysia; aqlima@upm.edu.my (S.A.A.); noorazmi@upm.edu.my (N.A.S.)

**Keywords:** microalgae, pigment, commercial, biotechnology, applications, bioproduction, Scopus database, R studio, CiteSpace

## Abstract

Phycobiliproteins are gaining popularity as long-term, high-value natural products which can be alternatives to synthetic products. This study analyzed research trends of phycobiliproteins from 1909 to 2020 using a bibliometric approach based on the Scopus database. The current findings showed that phycobiliprotein is a burgeoning field in terms of publications outputs with “biochemistry, genetics, and molecular biology” as the most related and focused subject. The Journal of Applied Phycology was the most productive journal in publishing articles on phycobiliproteins. Although the United States of America (U.S.A.) contributed the most publications on phycobiliproteins, the Chinese Academy of Sciences (China) is the institution with the largest number of publications. The most productive author on phycobiliproteins was Glazer, Alexander N. (U.S.A.). The U.S.A. and Germany were at the forefront of international collaboration in this field. According to the keyword analysis, the most explored theme was the optimization of microalgae culture parameters and phycobiliproteins extraction methods. The bioactivity properties and extraction of phycobiliproteins were identified as future research priorities. *Synechococcus* and *Arthrospira* were the most cited genera. This study serves as an initial step in fortifying the phycobiliproteins market, which is expected to exponentially expand in the future. Moreover, further research and global collaboration are necessary to commercialize phycobiliproteins and increase the consumer acceptability of the pigments and their products.

## 1. Introduction

Phycobiliproteins are indispensable photosynthetic accessory pigments responsible for light-harvesting in blue-green algae, cyanelles, cryptomonads, and red algae [1]. They are water-soluble pigments made up of proteins and covalently bound phycobilins [2,3]. Phycobiliproteins are primarily derived from blue-green algae and constitute up to 50% of total cellular proteins [4]. Phycobiliproteins are divided into three major types based on their absorption peak and structure: phycocyanin, allophycocyanin, and phycoerythrin [5]. Phycocyanin is the major phycobiliprotein, followed by phycoerythrin and allophycocyanin that were found in blue-green algae [5,6]. On the other hand, phycoerythrin is the dominant phycobiliprotein in most of the red algae, Rhodophyceae [7]. Besides, a few species of cryptophytes contain phycobiliproteins, and each cryptophyte usually has only one type of phycobiliproteins [8].

In recent decades, algae bioactive compounds or metabolites, including pigments such as phycobiliproteins, have sparked increasing interest [1,9]. Phycobiliproteins have been widely utilized in various fields, owing to their biological and pharmacological characteristics [10,11,12]. For example, phycobiliproteins’ non-carcinogenic and non-toxic properties have led to their usage in the feed, food, and cosmetics industries. Furthermore, phycobiliproteins have been employed as a surrogate of synthetic colorants [13,14]. Nowadays, phycocyanin is the primary market target. Its use has been expanded since its approval by the Food and Drug Administration (FDA) in the United States of America (U.S.A.) (https://www.foodbusinessnews.net/articles/2782-f-d-a-approves-natural-source-as-blue-color-in-candy-gum (Assessed date: 30 May 2021)). According to Future Market Insights, the overall market of phycobiliproteins for 2018 is USD 112.3 million, and it is expected to double this number in 2028 [1]. In the aquaculture industry, phycocyanin has been utilized as a feed supplement in shrimp, fish, or ornamental fish as it contains high nutrients and enables to increase the skin color [15].

Alan Pritchard proposed bibliometrics in 1969, which has been utilized in several subsequent research throughout the world [16,17,18,19]. Nowadays, it has grown into an indispensable instrument to analyze the current trends in scientific publications. Furthermore, it could serve as a possible guide for current and future research in certain fields or disciplines. VOSviewer, Gephi, CiteSpace, and Bibexcel are examples of frequently used bibliometric tools that construct keyword networks, complex social networks, knowledge mapping, and bibliometrics, respectively [19,20,21,22,23]. The ideal approach to measure and analyze worldwide scientific production and qualitative data may produce functional indices to recognize the present status and future potential within a specific research field or area [24].

Recently, bibliometric evaluations on microalgae research, microalgae biofuel, and even microalgae-derived pigments have been published [9,17,25]. Interestingly, although these publications addressed algae or metabolites produced from algae, there is no exhaustive bibliometric evaluation on phycobiliproteins [9,25]. Hence, this study aimed to provide a comprehensive worldwide research status and emerging trends of phycobiliproteins study via analyzing the global publishing landscape on phycobiliproteins research from 1909 to 2020. Herein, the primary information and the specific performance of publications related to phycobiliproteins research were retrieved from the Scopus database and analyzed thoroughly. Furthermore, keyword analysis over different periods was performed to highlight the directions and trends of phycobiliproteins research, thus raising the awareness of potential gaps in scientific collaboration.

## 2. Bibliometric Analysis

### 2.1. Methodology

The methods applied in this study were based on the work of Qi et al. [19] and Lim et al. [20], with modifications. Structured literature reviews were completed in three stages: identifying appropriate search terms, retrieving the content, and performing the analysis [26]. In this review, this three-step process was used for collecting data and detailed assessment.

#### 2.1.1. Data Collection

The information of scientific publications was collected from the Elsevier Scopus database (retrieved on 12 April 2021). A comprehensive search was performed using “phycobiliprotein* or phycocyanin* or phycoerythrin* or allophycocyanin*” as the search query of (TITLE-ABS-KEY) from 1909 to 2020 to compile a bibliography of all publications whose topics are related to phycobiliprotein*, phycocyanin*, phycoerythrin*, and allophycocyanin*. The use of an asterisk at the end of a word ensures that any terms with the same root are included in the search result. Data from 2021 were not included in this analysis for data consistency. Due to the limited number of documents that can be retrieved from the Scopus database per single search (maximum: 2000 documents), the data were collected over shorter periods (i.e., ten years) using the same search query. These retrieved data from 1909 to 2020 were combined prior to the bibliometric analysis. This search yielded a total of 8296 global publications; it should be noted that the results might vary if different search parameters are used.

#### 2.1.2. Refinement of the Search Results

Refinement of search results was conducted to exclude irrelevant articles and enhance the accuracy of bibliometric analysis for reliable review [18]. Hence, each publication from the search results was properly filtered by manual inspection while those publications unrelated to the phycobiliproteins topic were eliminated.

#### 2.1.3. Bibliometric Analysis

The obtained data were analyzed by using the open-source RStudio software (www.rstudio.com), version 4.0.5 (accessed on 30 April 2021), along with the bibliometrix R-package [27]. The information related to journal impact factors (IFs) and the *h*-index of each journal were obtained from the Journal Citation Reports (JCR) in 2020. The number of total citations was extracted from Scopus. VOSviewer (Leiden University, Netherlands), a software created by van Eck and Waltman, was used to construct and visualize a knowledge map of the co-occurrence network [28]. The keyword co-occurrence was performed to find the most often used keywords that related to phycobiliproteins. The study provided three options, namely “author keywords”, “index keywords”, and “all keywords”, with “all keywords” being selected; then, “full counting” was applied as the counting method. A minimum number of keyword occurrences was set to 20, and a manual inspection was carried out to remove unrelated keywords [9]. CiteSpace.5.7.R5W was also applied to analyze co-occurring keywords with different visualizations [29]. The research hotspots and frontiers in phycobiliproteins were assessed using this software by the burst detection analysis.

### 2.2. General Characteristics of Research Publications

A total of 8296 documents from 2214 different sources and 22,871 authors were listed during 1909–2020 (Table 1). The authors of multi-authored documents were 22,540, while the authors of single-authored documents were 331. The average years from publications on phycobiliproteins is around 16.7 per year, with each publication receiving an average of 30.88 citations and 1.85 citations per year. The annual growth rate of scientific production is 9.00%.

#### 2.2.1. Publication Types and Languages

**Figure 1:**
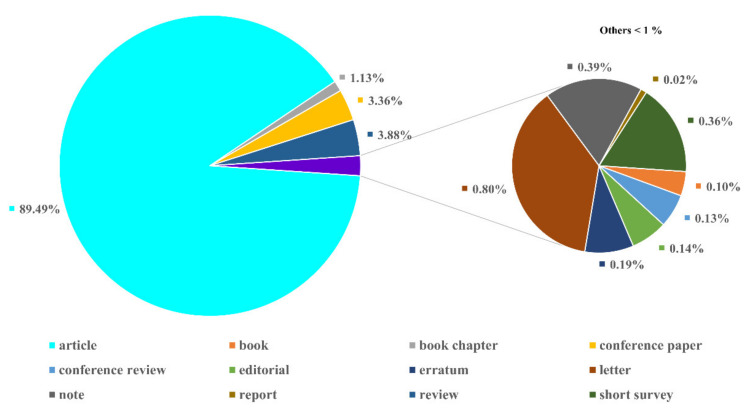
Distribution of phycobiliprotein publication types.

A total of 8296 publications were classified into 12 document types. The majority of the publications were identified as articles, forming 89.49% (7424 papers) of the total documents (Figure 1). Review publications accounted for 3.88% (322 documents), followed by conference papers and book chapters, with each accounting for 3.36% (279 documents) and 1.13% (94 documents), respectively. Other documents demonstrated a lower percentage of occurrence among the publications type. These included letters, with 0.80% (66 documents), notes, with 0.39% (32 documents), short surveys, with 0.36% (30 documents), erratum, with 0.19% (16 documents), editorials, with 0.14% (12 documents), conference reviews, with 0.13% (11 documents), books, with 0.10% (8 documents), and reports, with 0.02% (2 documents).

**Figure 2:**
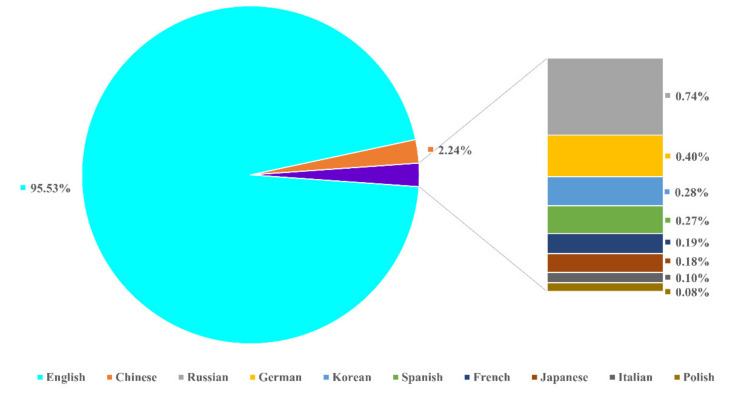
Distribution of languages used in phycobiliproteins publications.

The majority of the publications were in English (95.53%), while 2.24% of the publications were printed in Chinese (Figure 2). Publications in other languages accounted for 2.23%, consisting of Russian (0.74%), German (0.40%), Korean (0.28%), Spanish (0.27%), French (0.19%), Japanese (0.18), Italian (0.10%), and Polish (0.08%).

#### 2.2.2. Subject Categories of Publications

A total of 8296 publications from 1909 to 2020 covered 28 subject areas. Biochemistry, genetics, and molecular biology ranked the top, with 26.00%, among the other subject areas (Table 2). This was followed by agricultural and biological sciences, with 14.60%, medicine, with 12.60%, immunology and microbiology, with 9.10%, chemistry, with 7.00%, environmental science, with 5.90%, and chemical engineering, with 4.00%. Engineering, pharmacology, toxicology and pharmaceutics, and physics and astronomy accounted for 3.32%, 3.30%, and 2.80% among the other subject areas, respectively.

#### 2.2.3. Sources of Publications

Phycobiliproteins publications were distributed in 2214 different sources. The top 10 sources published 1083 articles, accounting for 13.07% of all publications (Table 3). Journal of Applied Phycology (publisher: Springer) ranked the top with 184 publications (2.22%), 4122 total citations, and an impact factor of 3.215. This was followed by the Journal of Biological Chemistry, which owned 137 publications (1.65%) with a 5.157 impact factor and the highest total citation (6672) among these top ten sources. Journal of Phycology was rated third with 127 publications (1.53%) and an impact factor of 2.923. Proceedings of the National Academy of Sciences of the U.S.A. possessed the highest impact factor (11.205) and *h*-index (699). Furthermore, 70.00% of the ten most prolific sources performed relatively well on the impact factor, ranging from 3 to 10, while the *h*-index value of all top ten sources was more than 90, except Cytometry. Based on the journal impact factor (JIF) ranking, all these top ten journals were above rank Q3, with 50% of the journals classified as rank Q1. Besides, Springer and Wiley were the most prolific publishers among the top ten most productive sources.

**Figure 3:**
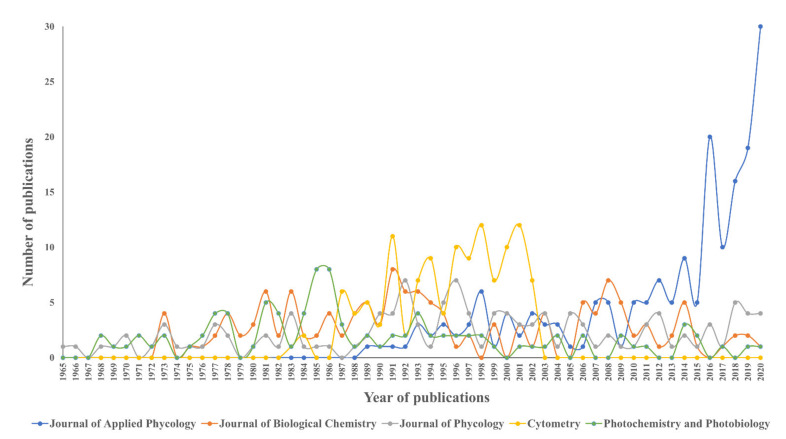
The trend of major five sources on phycobiliproteins publications from 1965 to 2020.

The research output of the top five journals displayed a growing fluctuation trend from 1965 to 2020 (Figure 3). Among the five journals, the Journal of Phycology was the journal that first published a document related to phycobiliprotein in 1965. The number of publications of the Cytometry has outperformed those in the other journals from 1996 to 2002, but this journal was discontinued by 2002 and published as Cytometry Part A and Cytometry Part B from 2003 onwards. The number of publications of Photochemistry and Photobiology dropped after the peak in 1985 and 1986, followed by a slower-growing trend towards 2020. The Journal of Applied Phycology was the fastest-growing journal between 2015 and 2020, despite a minor decline in 2017. The other four journals displayed a slightly slower trend in phycobiliproteins research when approaching 2020.

### 2.3. Specific Performance of Research Publications

#### 2.3.1. Annual Publication Trend

**Figure 4:**
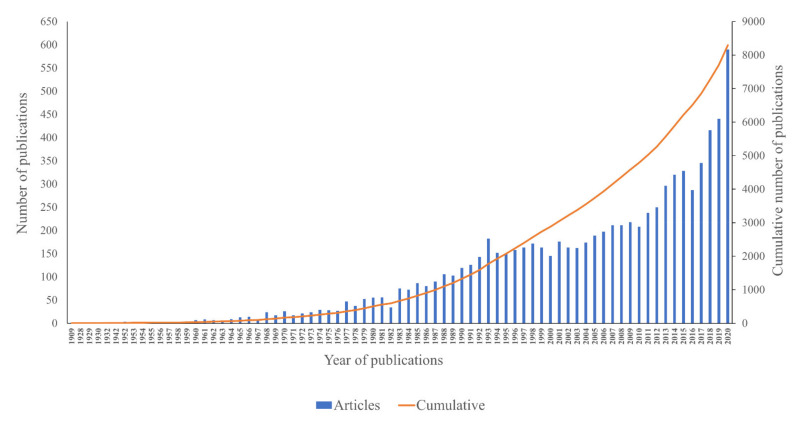
The annual and cumulative numbers of research publications on phycobiliproteins from 1909 to 2020.

The first publication was in 1909, according to the Scopus database (Figure 4). The number of publications per year remained below ten before 1964. The number of publications reached 182 in 1993, and the total publications were 1775 documents. Although there was a fluctuation in the trend of publications after 1977, a slightly growing trend was observed until 2015. There were 328 publications produced in 2015, followed by a slight drop in 2016 (287 publications). At this time, a total of 6505 total publications were accumulated. After that, a conspicuous increase was identified from 2017 to 2020, with a peak of 590 publications in 2020.

#### 2.3.2. Analysis of Growth Trend

Between 1909 and 1960, the total number of publications was less than 100, the total number of authors and references was less than 100, and the total number of citations (until 2020) was less than 1000 (Table 4). However, the total number of publications, the average number of authors, and references per publication have increased gradually in the following years. From 1961 to 1970, the average number of authors per publication decreased from 1.39 to 1.21. Then, the number slightly increased between 1971 and 1980, and before reaching 3.99 between 2011 and 2020. The number of citations (until 2020) and the average number of citations per publication declined from 81,576 to 57,738, and 42.73 to 16.45, respectively, between 2011 and 2020.

#### 2.3.3. Highly Cited Publications

Out of 8296 publications, 7020 publications received less than 50 citations, while 811 publications received 51–100 citations. There were 446 publications with citations ranging from 101 to 500, and 13 publications with citations ranging from 501 to 1000. A total of six publications received between 1001 and 2000 citations, while only four publications received more than 2000 citations. The article published by Huang et al. [30] in the Journal of Agricultural and Food Chemistry garnered the most citations (4010), in which the authors reviewed the chemical principles of antioxidant capacity assays (Table 5). Spolaore et al. [31] discussed the applications of microalgae and published in the Journal of Bioscience and Bioengineering. This article was ranked second with 2574 total citations. Cao et al. [32] discussed the antioxidant and prooxidant behavior of flavonoids and published it in Free Radical Biology and Medicine. This article was rated third in the top ten cited publications. Ou et al. [33] developed an improved method of oxygen radical absorbance capacity (ORAC) assay, which the article was cited for 2024. Jones et al. [34] explored the new approach to protein fold recognition in their article, which was ranked last in the top ten cited publications, with 1035 total citations. The journals with the most cited publications (Table 5) were not found in the top ten productive sources of phycobiliproteins publication.

#### 2.3.4. Performance of Publications by Countries

The U.S.A. led the total of publications in the study of phycobiliproteins, with 27.00% of publications output (Table 6). Each of the top prolific countries published at least 200 publications. Before 1960, the U.S.A., Japan, and the United Kingdom contributed eleven, six, and three publications, respectively; this number progressively increased over the following six stages with a slight drop between 2001 and 2010. The U.S.A. contributed 538 and 470 publications during the fifth (1991–2000) and sixth period (2001–2010), making it the most productive country. The number of publications produced by China increased profoundly from eight publications in the fourth period (1981–1990) to 791 publications in the last period (2011–2020), surpassing the U.S.A. Spain joined the phycobiliprotein field later than the other countries. However, Spain contributed more publications in the last stage compared to the United Kingdom, Italy, and Canada. Overall, the number of publications of the top ten productive countries increased at different rates over the seven periods.

**Figure 5:**
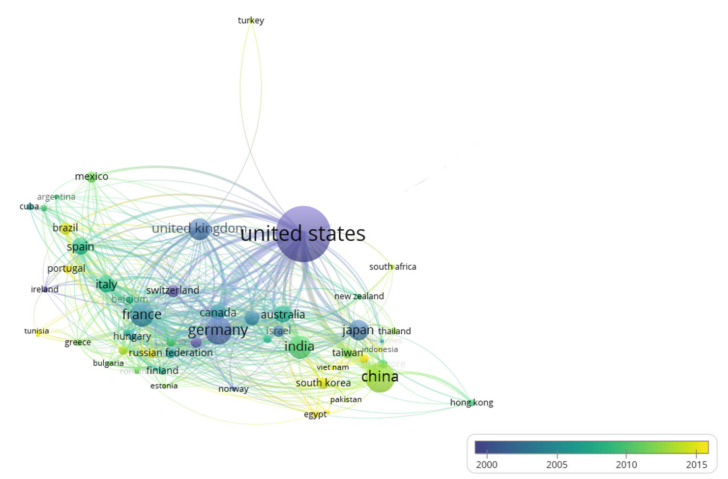
The overlay visualization map for the co-authorship between countries in phycobiliproteins research (minimum occurrences: 10).

Worldwide collaborations on phycobiliproteins study were constructed using VOSviewer (Figure 5). Each dot denotes a country, while the size of the dot illustrates the country’s cooperative publishing capabilities, followed by the lines between dots that display the number of cooperative publications, which indicates the countries’ collaborative relationships (link). Each link has a strength, represented numerically by a positive value—the greater this number, the stronger the link. The overall connection strength denotes the number of publications in which the collaborated countries appear together [41]. The U.S.A. and most of the European countries, such as Germany (494 of total link strength), France (272 of total link strength), and the United Kingdom (262 of total link strength), demonstrated the most international collaborations. In contrast, most of the Asian countries, including India (104 of total link strength), Malaysia (30 of total link strength), Singapore (28 of total link strength), and Thailand (21 of total link strength), were lacking in international collaborations. With a total link strength of 771, the U.S.A. and Germany were the largest distributors in the phycobiliproteins research. Furthermore, Figure 5 depicts research collaborations between the top prolific countries (represented by purple and blue nodes) and researchers from the recently involved countries (represented by green and yellow nodes). The overlay visualization feature is used to explore the emerging collaborations in phycobiliprotein research. The scores were assigned to each item collected for analysis in this type of visualization. While searching for emerging collaboration in this study, the date (year) of collaboration represented the score [42]. In the visualization, the purple color indicated the lowest score (the earliest collaborations) and the highest item density in the purple-blue-green-yellow scheme, while the yellow color displayed the highest score (the most up-to-date collaborations) and the lowest item density. The more intense the purple, the higher the number of citations per article. On the other hand, the more intense the yellow, the fewer the number of citations per article [18]. The light green color of China indicates a relatively low average number of citations per article, which was no more than 20, despite having the most articles. Similarly, India poorly performed on article citations. Publications of the United Kingdom and France were not numerous but were highly cited (more than 40 citations per publication).

#### 2.3.5. Performance of Publications by Institutions

A total of 6861 institutions have contributed to the study of phycobiliproteins. Among these institutions, 266 (3.21%) produced more than ten publications. Chinese Academy of Sciences contributed the highest number of publications (352 articles: 4.24%) among the top ten institutions (Table 7). The top ten rankings were dominated by Asian and European institutions (three China, two U.S.A., two Russia, one French, one Germany, and one Japan). Although the U.S.A. was the most prolific country in the phycobiliproteins research, only two institutions from the U.S.A. were listed in the top ten institutions. Although the three China institutions joined the phycobiliproteins study later, their number of publications increased dramatically in the last two phases, allowing them to be in the top ten productive institutions. The extraordinarily high number of total citations and average total citation per publication by the University of California, Berkeley (U.S.A.) demonstrated that the U.S.A. was still the most prolific country in phycobiliproteins research. Lomonosov Moscow State University was positioned last in the top ten productive institutions.

#### 2.3.6. Performance of Publications by Authors

From 1909 to 2020, 22,871 authors contributed to the research on phycobiliproteins. The top 10 prolific authors were affiliated with the following countries: the U.S.A. (four authors), China (two authors), Germany (one author), Israel (one author), India (one author), and Russia (one author). Glazer, Alexander N. from the University of California in the U.S.A. led the top ten productive authors with 118 publications (1.42%; *h*-index: 65) since 1971 (Table 8). He had the most publications (57) during the period 1981–1990. Scheer, Hugo (Germany) came in second with 89 publications (1.07%; *h*-index: 49), while Bryant, Donald A. (U.S.A.) came in the third with 64 publications (0.77%; *h*-index: 72). Berns, Donald S. from the Israel Ministry of Health, Israel, was placed fifth with 59 publications (0.71%). MacColl, Robert from the Wadsworth Center for Laboratories and Research, U.S.A., was the seventh among the top ten prolific authors with 52 publications (0.63%; *h*-index: 25). Madamwar, Datta B. from India and Bekasova, Olga D. from Russia were ranked as eighth and ninth productive authors with 45 (0.54%) and 40 (0.48%) publications, respectively. Two authors from China, Qin, Song and Zhao, Kaihong, were ranked as fourth and sixth among the top ten authors with 62 (0.75%) and 55 (0.66%) publications, respectively.

### 2.4. Main Research Hotspot and Trends

#### 2.4.1. Keywords Analysis

**Figure 6:**
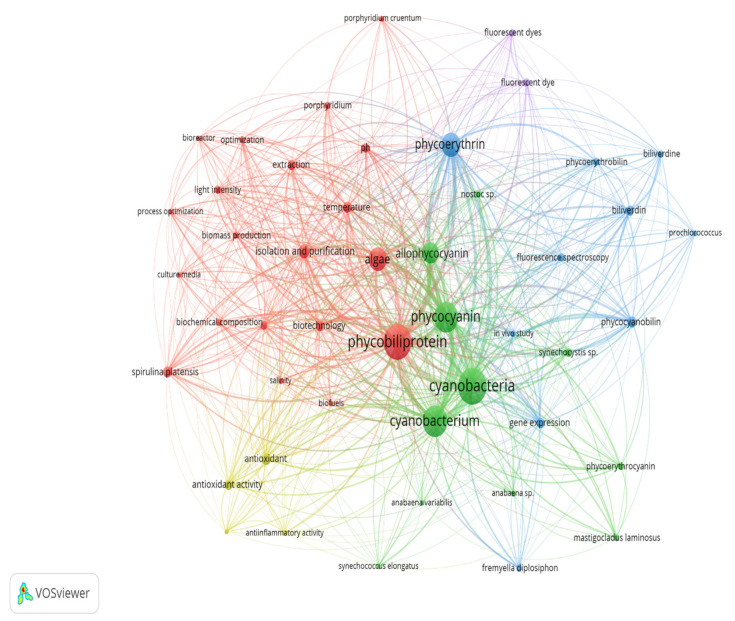
The bibliometric map based on total co-occurrence analysis with network visualization from 1909 to 2020 (minimum occurrences: 20).

The co-occurrence of keywords (co-word) was analyzed to allow researchers to postulate the critical topics and knowledge structure in the phycobiliproteins research fields. Co-words are pair of keywords that appear together in a publication, and the analysis was conducted based on the frequency of co-occurrence. The keyword analysis began after the key terms unrelated to phycobiliprotein were eliminated. Each circle represents a keyword with at least 20 occurrences, and its size corresponds to the number of occurrences (Figure 6). The larger the keyword circle, the more frequent the occurrences of the keyword. Clusters identify a group of items related and are represented on the map with different colors.

Although 879 terms met the predefined criteria in the phycobiliprotein research, only 52 keywords were deemed relevant and analyzed in the keyword co-occurrence network. The results showed that the co-words were mapped into five major clusters, implying that five different research themes could be derived (Table 9).

#### 2.4.2. Analysis of Research Trend–Burst Detection Analysis

**Figure 7:**
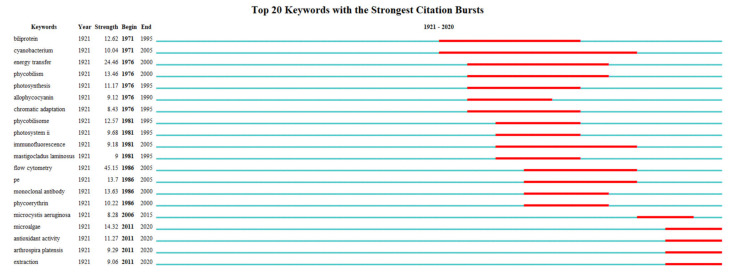
The top 20 keywords with the strongest citation burst.

An apparent citation burst strength of the keywords in phycobiliproteins research was displayed from 1921 to 2020 (Figure 7). The citation burst began with biliprotein and cyanobacterium in 1971 with a burst strength of 12.62 and 10.04, respectively. This was followed by energy transfer, which first appeared in 1976 with a burst strength of 24.46. Cyanobacterium had the most extended burst, in which this keyword was cited for 35 years. Flow cytometry had the greatest burst strength (45.15). The citation bursts of biliprotein, energy transfer, phycobilism, and immunofluorescence lasted for 25 years. The citation burst of each following keyword: photosynthesis, allophycocyanin, chromatic adaptation, phycobilisome, photosystem II, *Mastigocladus laminosus*, flow cytometry, pe (phycoerythrin), monoclonal antibody, phycoerythrin, and *Microcystis aeruginosa* were cited at least for 20 years. Based on the burst detection analysis, microalgae, antioxidant activity, *Arthrospira platensis,* and extraction emerged as new research areas in recent years.

#### 2.4.3. Analysis of Research Trend: Algae Genera

**Figure 8:**
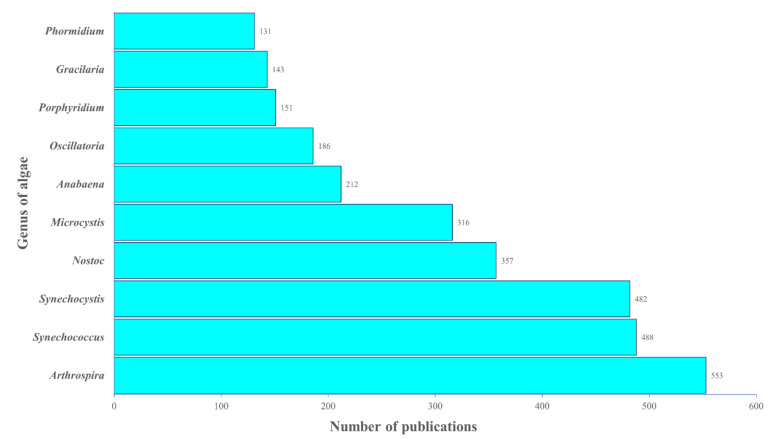
The top ten genera of algae related to the phycobiliprotein study.

Thirty-eight algal genera appeared in the titles, abstracts, or keywords of the 8296 articles. Blue-green algae were accounted for 80% of the top ten listed genera, whereas red algae were responsible for the remaining 20% (Figure 8). *Arthrospira* genus appeared in 553 articles, which was ranked first in the top ten genera. *Synechococcus* (488), *Synechocystis* (482), *Nostoc* (357), *Microcystis* (316), and *Anabaena* (212) were the species that were present in at least 200 articles. *Phormidium* (131) and *Gracillaria* (143) were ranked underside in this top ten genera, with approximately four times fewer appearances in publications than *Arthrospira.*

## 3. Overview of Previous Phycobiliprotein Research

**Figure 9:**
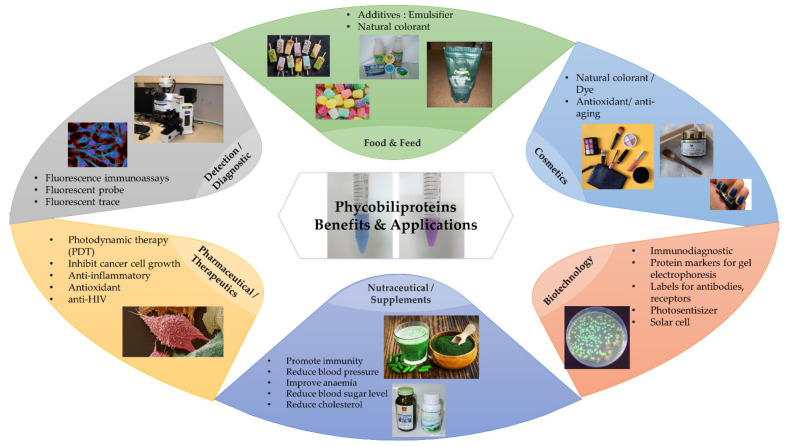
The benefits and applications of phycobiliproteins in different industries (adapted from [1,50,51,52,53]).

To date, several researchers have published critical summaries and valuable insights on different aspects of phycobiliproteins study [1,5,43]. For example, Manirafasha et al. [5] outlined the properties of phycobiliproteins, the mechanism regulating phycobiliproteins biosynthesis, and the enhancement of phycobiliproteins production. Liu et al. [44] discussed medical applications of phycocyanin extracted from *Spirulina platensis*. In addition, Li et al. [45] provided a critical review on the molecular structure, production, and applications of phycobiliproteins. Ming et al. [43] reviewed the methods for enhancing phycocyanin and phycoerythrin production yield and chemical stability. Moreover, Kuddus et al. [46] outlined the C-phycocyanin production and biotechnological applications. Sui [47] summarized the structure of phycobilisomes, while Silva et al. [48] reviewed the mechanisms of action and multidrug resistance of phycocyanin in cancer. However, there is still a paucity of information on the present, current, and future research trends of phycobiliproteins. Following the rapid growth of scientific research, bibliometric analysis is now frequently applied to analyze the research dynamics in several fields. This type of study focuses on the quantitative assessment of publications as well as the related bibliographic citations and proxies [49]. Garrido et al. [25] performed bibliometric analysis to analyze and provide a global overview of microalgae research. Furthermore, Samara et al. [9] conducted a ten-year bibliometric analysis on microalgae-derived pigments. Phycobiliproteins are well-known natural pigments with several potential benefits and applications (Figure 9). These pigments have appeared as an emerging topic in various fields [3,5,50]. Therefore, a bibliometric analysis is necessary to comprehend the current trend, identify the challenges, and discover future research opportunities.

### 3.1. General Characteristics of Research Publications in Phycobiliproteins Study

According to Scopus database, the first study on phycobiliproteins began in 1909 [54]. According to Figure 1, phycobiliproteins research has been disseminated in 12 different types of publications. This demonstrates that phycobiliproteins research embraces a variety of thematics [9]. The highest percentage of documents published are journal articles. This revealed that numerous researchers contributed novel ideas to explore and study phycobiliproteins in depth. Review occupies the second position in the publication type as review underlying the essence development of a field [25]. The most widely applied language in publications was English (Figure 2). This is because articles that appeared in the international journals are mainly in English [25]. Besides, it is essential to note that a single publication may be published in more than one language.

Each publication indexed by Scopus was assigned with at least one subject area. Phycobiliproteins research could be categorized into 28 subject areas. Biochemistry, genetics, and molecular biology ranked first among the top ten most productive subject areas (Table 2). This indicates a strong scientific interest in studying the process or mechanism underlying phycobiliproteins production at the molecular level, as well as their structures, properties, or gene expression of phycobiliproteins biosynthesis [55,56]. The growing interest in phycobiliprotein production, purification, and uses led to the predominance of the agricultural and biological sciences subject area [57,58]. Phycobiliproteins’ bioactivities and applications of phycobiliproteins in the environment and medicine were the topics of the subject area of environmental science and medicine [1,5]. “Immunology, microbiology and chemistry” were ranked high in the top ten due to the breakthrough of fluorescent properties of phycobiliproteins and their potential application, such as labels in immunoassays [59,60,61]. Indeed, phycobiliprotein study requires contributions from various research areas, making it multidisciplinary. However, a modest number of publications (less than 20) were found in economics (16), business and management (13), and social sciences (12). This indicates that the current phycobiliprotein applied research is still immature, even though commercial applications for phycobiliproteins have been developed [62]. Hence, more research into the economic viability and consumers’ acceptance of phycobiliproteins is needed.

The phycobiliprotein research was published in 2214 sources from 1909 to 2020. Five Q1 journals, one Q2 journal, and three Q3 journals were among the top ten prolific sources (Table 3). The top ten productive sources accounted for 13.07% of all phycobiliprotein publications. This signifies that one-fifth of the researchers selected high-impact or high-ranking journals to publish their novel phycobiliproteins study. Springer and Wiley occupied three places in the top ten journals, respectively. This could be due to the reputation of two publishers that have been established for more than 140 years (https://en.wikipedia.org/wiki/Springer_Publishing) (Assesed date: 20 June 2021) (https://en.wikipedia.org/wiki/Wiley_(publisher)) (Assesed date: 20 June 2021). Besides, they also provide scholars with access to millions of peer-reviewed, open-access scientific documents. The citation report of each journal was generated based on the papers chosen for this study to compute the *h*-index [49]. The *h*-index was used to evaluate a country’s, institute’s, or researcher’s contribution. It is defined as the number of articles with citation numbers higher than or equal to *h*. In addition, the *h*-index does not only represent the actual production, but also the apparent influence of a group’s or a scholar’s published work [18]. Previous research also found that the *h*-index has a higher predictive potential than the total number of articles published, total number of citations, and average citations per publication [63]. The proceedings tend to have a high *h*-index. Furthermore, each journal’s impact factor value was determined using the Journal Citation Reports (JCR) in 2020. The JCR impact factor value and the *h*-index value of a source could serve as good indicators in predicting the impact and number of citations received by journals. The JCR impact factor value can be used as an index for researchers to postulate suitable journals when dealing with phycobiliproteins studies [25]. Both indexes have the potential to impact certain authors’ judgments when it comes to select journals that are appropriate for their most novel and notable work [64]. Regarding the indexes in this review, all the top five sources were ranked Q1 and Q3 based on the JCR impact factor ranking except for the Cytometry. The impact factor of Cytometry was no longer available in the JCR ranking, as the journal was published as Cytometry Part A and Cytometry Part B from 2003 onwards. For both indexes, Proceedings of the National Academy of Sciences of the U.S.A. ranked the highest, implicating that it has the best quality source in the phycobiliprotein field. Although both indexes of the Journal of Applied Phycology were slightly low, the increase in the number of publications and positive evolution in scientific production over the last five years has corroborated the Journal of Applied Phycology as one of the potential quality sources in the phycobiliprotein field.

### 3.2. Annual Publication Trend in Phycobiliproteins Research

In terms of annual publication trends, interest in phycobiliproteins research began in 1909. Since then, annual publications have steadily increased (Figure 4). Dramatic elevation of publications was observed from 2017 to 2020, which could be attributed to a more extensive involvement of China and India in phycobiliprotein research. The rising number of publications suggests that there are still many undiscovered interesting topics related to phycobiliproteins. Therefore, it is predicted that the annual number of publications will continue to rise. Over the past 40 years, research on phycobiliproteins has continued to expand, while its relation and collaboration have become increasingly active. Overall, the average number of authors, average number of references, and average number of citations per publication increased with the increase in the number of publications (Table 4). However, there were contrasts at particular periods. For example, the number of citations until 2020 per publication decreased with the increasing number of publications between 2001 and 2010. This is because the majority of these publications are not freely available and need payment to access the information in them. If an article is published in an open-access publication, it will acquire more citations [64]. As of 2020, only 2614 (31.51%) articles have been published as open access.

Findings from highly cited publications are very impactful since they reflect scientific advancement recognition, give novel and vital insights, and provide a historical perspective on scientific advancement [18]. The first- and second-ranked in the frequently cited articles were reviews (Table 5). These reviews provide an overview of fundamental knowledge of phycobiliproteins bioactivities and applications. Among them, the review that described the chemical principles of antioxidant capacity assays owned the most total citations [30]. The second review summarized the commercial applications of microalgae [31]. This might be attributed to the growing market for phycobiliproteins and other pigments, which piques the interest of researchers in advancing the development for each application and characteristic of microalgae-derived pigments [1]. The third-ranked paper was published by Cao et al. [32], and studied the antioxidant and prooxidant behavior of flavonoids as well as the related activity–structure relationships. The fluorescent characteristics of phycobiliproteins aroused the interest of researchers, which led these fluorescent properties to be exploited into a variety of fields [59,65]. For example, Ou et al. [35] used fluorescent phycobiliproteins technology to develop an improved oxygen radical absorbance capacity assay (ORAC). Meanwhile, the fluorescent properties of phycobiliproteins, especially those exploited from the phycoerythrin, had been utilized in studies of Braud et al. [36], Lyons et al. [37], Chee et al. [39], and Cao et al. [40]. As a result, it is possible to infer that the top ten most cited articles could demonstrate the progress and shift of researchers’ interest toward the applications and beneficial properties of phycobiliproteins.

### 3.3. Country Involved in Phycobiliproteins Research

A total of 86 countries are involved in producing publications on phycobiliproteins; thus, phycobiliproteins have become a research focus worldwide. Each of the top ten countries contributed more than 200 publications, suggesting that these countries play a significant role in phycobiliprotein research, and are constantly committing innovative ideas to that purpose (Table 6). The U.S.A. and China contributed around 41.19% of publications. This indicated that these two countries were the key players in the advancement of phycobiliproteins research. During 2011–2020, the number of publications in China outnumbered the first-ranked (the U.S.A.). It is believed that China has strengthened international collaboration with other countries (Figure 5). However, the U.S.A. continued to have the most measurable impact on phycobiliproteins research, as evidenced by the highest *h*-index, owing mostly to the earlier publications. Although China was ranked second in the article output, the impact of its publications was relatively low based on the average number of citations per article. Germany has fewer publications than China, but its total citations and *h*-index are higher. The same can be said for the fourth- to tenth-ranked countries in the top ten most productive countries. Although the research by China had improved in terms of quantity, the quality of publications still requires polishing [19].

### 3.4. International Collaboration in Phycobiliproteins Field

International collaboration has emerged as an essential fraction of scientific study to improve economic growth and advancement of society [66]. Around 46.20% of the countries showed a total link strength greater than 50, indicating a strong collaboration among international countries in studying phycobiliproteins. The U.S.A. and most European countries have more international scientific collaboration than Asian countries. Thus, this resulted in more European countries frequently occupying the top ten list of productive countries. At the same time, there was a gap to fill in terms of exchanging of researchers and cooperation based on Figure 5. For example, the U.S.A. (the major international cooperating country with the most publications) did not collaborate with Malaysia or Thailand throughout the last ten decades. A large number of foreign postgraduates/visiting scholars, diversity of research partners, and robust research funding were all the likely variables that contributed to the dynamics of international collaboration. It is also crucial to have a flexible research policy to support the long-term viability of international cooperation [64].

The scientific research capabilities and exploratory atmosphere of the research institutions can be determined by analyzing the distribution of the research institutions where the authors work [19]. China, France, the U.S.A., Germany, Japan, and Russia had the top ten most productive institutions (Table 7). China, the U.S.A., and Russia each had at least two institutions on the list. This suggests that these countries have significant research capabilities and have invested more than other countries in phycobiliproteins research. Both Russian institutions (Russian Academy of Sciences and Lomonosov Moscow State University) were listed in the top ten research institutions, even though Russia was excluded from the top ten most productive countries. Based on Figure 5, Russia was denoted with the light green color dot, indicating that Russian scientists are still novices in international collaboration. Publications from Russian institutions began in 1964 and increased over the last two decades (Table 7). The high number of publications led both Russian institutions to be rated within the top ten prolific institutions. Most institutions began to focus on phycobiliproteins research in the fifth stage (Table 7). In addition, four universities from the top ten prolific institutions were listed among the world’s 100 best universities in the World University Rankings 2020: Lomonosov Moscow State University (ranking 84th), Ludwig-Maximilians-Universität München (ranking 63rd), The University of Tokyo (ranking 22nd), and University of California, Berkeley (ranking 28th) (https://www.topuniversities.com/university-rankings/world-university-rankings/2020 Assessed date: 25 June 2021)). This indicates that the world’s top institutions are interested in phycobiliproteins study.

Even though the U.S.A. led the world in the number of publications on phycobiliproteins research (over 2000 publications), no institution ranks the first in the top ten prolific institutions. This could be explained by the fact that two American institutions produced at least 100 publications on phycobiliproteins. Still, only one French, one Chinese, or one German institution could generate the same number of publications. The weight of research into phycobiliproteins study in the latter countries was primarily from a single institution—the Centre National de la Recherche Scientifique (CNRS) in France, the Chinese Academy of Sciences in China, and the Ludwig-Maximilians-Universität München in Germany. In the U.S.A., interest in phycobiliproteins is far more homogeneously distributed between research centers [25]. India was well ranked in terms of the number of publications, but their institution was not within the top ten prolific institutions. This condition is the same as in the U.S.A. Although the number of publications produced by Centre National de la Recherche Scientifique (CNRS) and the University of California, Berkeley was lower than those of the Chinese Academy of Sciences, it should be recalled that the Chinese Academy of Sciences has 124 branches; hence, a direct comparison may be biased [64]. Although the Chinese Academy of Sciences owned numerous subordinate research centers, such as the Institute of Oceanology, the Institute of Chemistry, and the Institute of Microbiology, its *h*-index was only ranked third [19]. In contrast, the University of California, Berkeley from the U.S.A. showed the highest average citations per publication and the highest *h*-index among the top ten institutions. This could imply that the University of California has been focusing on the quality of each publication instead of quantity. The benefits of international collaboration are also reflected in the top ten prolific author list (Table 8). The top ten productive authors were mostly from the U.S.A. and China, due to the stronger international collaboration of these countries with the other countries (Figure 5).

### 3.5. Research Trend in Phycobiliproteins Research

Keywords are the basis of bibliographic research of academic literature [67]. Keyword analysis indicates researchers’ emphasis on a specific study topic, making it an important component of bibliometric analysis [68]. The visualization network map (Figure 6) was created with VOSviewer to assess the occurrence relationships between the keywords collected from phycobiliprotein research articles [49]. Five clusters with different colors were determined in the keyword map, with a high degree of overlap (Table 9).

#### 3.5.1. Optimization of Cyanobacteria Cultivation and Phycobiliproteins Harvesting Process

**Figure 10:**
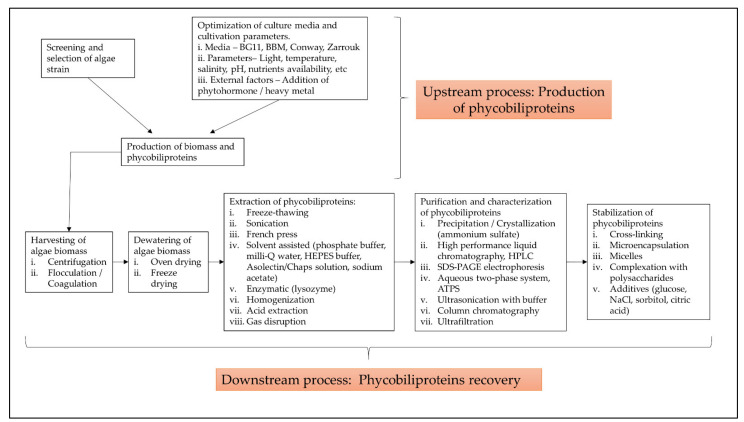
The process involved in production of high-purity phycobiliproteins (adapted from [5,43,74,75]).

The term “phycobiliprotein” was grouped with other 20 terms in cluster 1 (Figure 6, in red), which focuses primarily on process optimization of algae cultivation, algae biomass harvesting, and phycobiliprotein production. Successful cultivation technologies rely on algae species. In addition, phycobiliprotein production is also affected by cultural conditions and nutrition factors. These factors must be optimized to develop a feasible, sustainable, and economically viable culture system for algae [69,70]. The production of high-purity phycobiliproteins comprised a series of concomitant steps that included two main sequential processes, upstream and downstream processes, as shown in Figure 10. For high-quality phycobiliproteins, the optimum production conditions and parameters are required. Hence, it is critical to focus on each step of the phycobiliproteins production process to improve the accumulation of high-quality phycobiliproteins from each species [1]. Several studies have been carried out in depth for this purpose [1,2,71,72]. For example, Manirafasha et al. [5] reviewed the techniques to increase phycobiliprotein production, from algae strain selection to culture parameter optimization and phycobiliprotein extraction to phycobiliprotein purification. Furthermore, Begum et al. [73] discussed the effect of different drying methods on the production and purity of phycobiliproteins. In addition, Lo et al. [43] reviewed the procedure followed to increase phycocyanin and phycoerythrin production yield and stability.

#### 3.5.2. Classification with Structure

The term “cyanobacteria” was the central theme of the keyword map. It is part of cluster 2 (Figure 6, in green), which included 11 additional keywords. The classification of phycobiliproteins was the focus of this cluster. Phycobiliproteins are classified primarily by their absorbance spectrum properties: phycocyanin, PC (Amax = about 620 nm), phycoerythrin, PE (Amax = about 560 nm), and allophycocyanin, APC (Amax = about 650 nm) [1]. Each phycobiliprotein comprises a different polypeptide subunit (α and ß), containing covalently linked open-chain tetrapyrrole chromophores [3]. The chromophores, known as phycobilins, are covalently linked to the proteins via one or two thioether bonds to specific cysteine residues [76]. There are various structurally distinct phycobilin chromophores having distinctive spectroscopic characteristics that are also modulated by the phycobiliprotein quaternary structure [1]. The chromophores phycourobilin (PUB), phycobiliviolin (PXB), phycoerythrobilin (PEB), and phycocyanobilin (PCB) exhibit different absorbance maxima at around 498 nm, 568 nm, 535 to 567 nm, and 620 to 660 nm, respectively, when covalently linked to phycobiliproteins [76]. Furthermore, phycobiliprotein that exist as aggregates of heterodimers of alpha and beta subunits have several highly conserved amino acid residues necessary for αß heterodimer formation and chromophore binding [77]. Hence, it should be expected that some novel phycobiliproteins will be discovered throughout time. For example, Montgomery et al. [78] discovered a new and unique set of proteins that are most closely linked to allophycocyanin members of the phycobiliprotein superfamily. Each of these proteins are known as allophycocyanin-like (*Apl*) proteins. Novel chromophore types have been discovered in Cryptomonad phycobiliproteins [79,80]. The phycobiliproteins are further classified into many subtypes based on the chromophores’ number, combination, and position [79,80].

#### 3.5.3. Gene Expression of the Phycobiliproteins Biosynthesis Pathway

**Figure 11:**
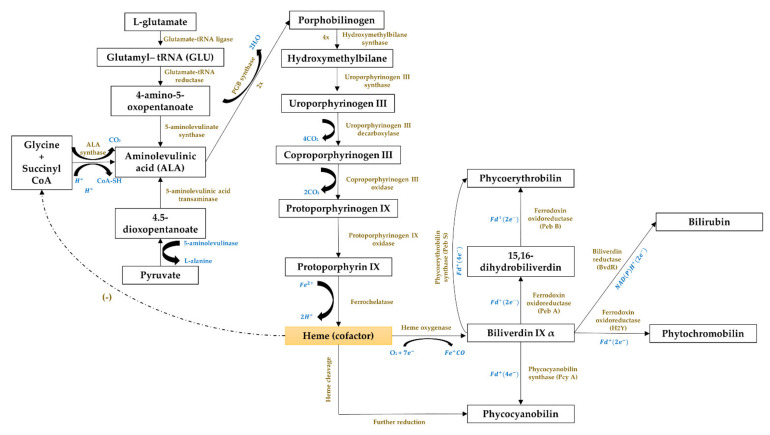
The biosynthesis pathway of phycobiliproteins (adapted from [85,86]).

The third cluster (Figure 6, in blue) focused on the gene expression of the phycobiliproteins biosynthesis pathway. The biosynthesis of phycobiliproteins occurs via transcription, translation, and posttranslational pathways, which included the synthesis of amino acids, proteins, and phycobilins, as well as the ligation of phycobilins formed to apoproteins during the posttranslational phase (Figure 11) [81,82]. Heme acts as a cofactor in various biological roles, including enzyme activity regulation, and represents the appropriate metabolic path of all bilin biosynthesis pathways [83]. Biliverdin is a common intermediate and precursor in the biosynthesis of bilins [84]. However, biliverdin sometimes will not be an intermediate if the reduction process happens before heme cleavages [84]. Manirafasha et al. [5] and Pagels et al. [1] summarized the mechanism of phycobiliprotein biosynthesis.

#### 3.5.4. Bioactivities and Applications of Phycobiliproteins

The theme of the fourth cluster (Figure 6, in yellow) was phycobiliprotein bioactivities. Natural sources of bioactive compounds are gaining popularity, as they can benefit humans [50]. The rising demand for natural bioactive molecules extracted using a simple approach has sparked the attention of researchers to explore unusual sources, including cyanobacteria [1]. Phycobiliproteins are highly valued natural products with various applications, including medicinal, nutraceutical, food, feed, and cosmetics (Figure 9). The research on phycobiliproteins bioactivities has grown in recent years, yet most of the research was focused on the bioactivities of phycocyanin [87,88,89]. For instance, Fernández-Rojas et al. [90] and Yu et al. [74] reviewed the bioactivities of phycocyanin. Furthermore, Pagels et al. [1] described the bioactivities from phycobiliproteins such as antioxidant capacity, anticancer, anti-inflammatory, anti-diabetes, antibacterial, anti-obesity, and neuroprotector agent. The fifth cluster (Figure 6, in purple) focused on the phycobiliprotein applications especially as the fluorescent dye. As summarized in Section 3 (overview of previous phycobiliprotein research), phycobiliproteins have been widely utilized as highly valuable compounds or natural products in various industries (Figure 9) [7,31,44,45,52].

### 3.6. Future Research Prospects in Phycobiliproteins Study

Research frontier refers to a growing trend in research theory and subject content that may be represented using burst keywords [91]. Kleinberg introduced the burst detection approach in 2002. Burst keywords are terms that suddenly increase within a short time [29]. It is possible to reveal the information that does not meet the frequency criteria but has informatics importance in academic advancement using the burst detection approach. It may be more practical and scientific to depict interaction and development trend of research frontiers by identifying hotspots change [19]. CiteSpace (a type of citation visualization software) was used to create the scientific knowledge mapping of burst detection to assess the research hotspots of phycobiliproteins [92,93]. Over time, the research emphases and orientations can be more directly represented by analyzing the changes in the most used author keywords in different periods. This study demonstrated that the earlier publications related to phycobiliprotein were its role in photosynthesis and energy transfer to chlorophyll, resulting in a citation burst of “energy transfer” and “photosynthesis” from 1976 (Figure 7) [62,74,94]. Phycoerythrin was widely explored due to its fluorescent properties [59,65]. Furthermore, the application of phycoerythrin and allophycocyanin in flow cytometry has gained prominence since 1986 [95,96]. The majority of the phycobiliproteins were identified in cyanobacteria, especially *Arthrospira platensis*. The bioactivity properties of phycobiliproteins (specifically antioxidant activity) have piqued the interest of researchers since 2011 and have remained a research frontier until now [1,5]. Economically sustainable and environmentally friendly phycobiliprotein extraction methods have also gained popularity and have helped broaden the consumer acceptability of cheaper and safer natural pigments [71,72,97,98].

Phycobiliproteins are mainly found in blue-green algae, yet could also be found in red algae, cyanelles, and cryptomonads [1]. Most researchers used blue-green algae for their phycobiliproteins research, whereas only two red algae genera out of ten were exploited (Figure 8). The recent studies on red algae investigated the structure of phycobilisome in *Griffithsia pacifica* and the structural basis of energy transfer in phycobilisome of *Porphyridium purpureum* [99,100]. The *Arhtrospira* genus was the most used model organism. The *Arthrospira* genus is broadly recognized for its high phycocyanin content [5]. In addition, *Arthrospira maxima* has been commercially utilized as a food since 1521 [101]. The oldest records of production of *Arthrospira* biomass for human consumption are from the Aztecs [102]. Furthermore, *Arthrospira* has also been exploited as a protein supplement by the Kanembu tribe of Africa near Lake Chad since 1940 [103]. *Synechococcus* is an unicellular and euryhaline cyanobacterium [104]. *Synechococcus* is the most plentiful (up to 10^5^ mL^−1^) and widespread picophytoplankton genus in the open ocean [105]. It is commonly used as a model organism to study cyanobacterial metabolism, particularly photosynthetic research, and has the potential for biotechnological uses [105,106]. It is also touted as a phycocyanin and phycoerythrin-rich genus [107]. Furthermore, it has a fast growth rate and an extraordinary resistance to high light irradiation [104]. Hence, these characteristics of *Synechococcus* favored most researchers in selecting this cyanobacterium for their study. On the other hand, *Synechocystis* and *Nostoc* genus are the other blue-green algae frequently employed in phycobiliproteins research due to their high nutritional values, and they are widely commercialized. *Synechocystis* has received attention in modeling studies and biotechnological applications due to a variety of characteristics including its fast growth, the potential to fix carbon dioxide into valuable products, and the relative simplicity of genetic modification [108]. Despite *Synechocystis*, the *Nostoc* genus is employed as a food and feed supplement in Mongolia, China, and South America [103]. *Nostoc commune* has long been recognized as a worldwide nutritious meal and traditional medicine [109]. A wide variety of notably pharmacological and protective physiological properties of the *Nostoc* genus aroused the attention of researchers [109]. On the other hand, the number of algae commonly claimed as toxic genera (*Microcystis, Anabaena, Phormidium, and Nostoc*) was lower than the nontoxic algae genera (*Porphyridium, Oscillatoria, Gracilaria, Synechocystis, Arthrospira, and Synechococcus*) (Figure 8). This indicated that more studies were focused on the benefits of cyanobacteria and their bioactivities. *Microcystis* and *Anabaena* are the most important toxic cyanobacteria bloom genera in terms of diversity, impact potential, and cascading ecological effects [110,111]. Although numerous microalgae species are available in various culture collections worldwide, only a minority have been thoroughly studied [25]. Strains such as *Haematococcus pluvialis* (main source of astaxanthin), *Dunaliella salina* (the major source of beta-carotene), and *Spirulina platensis* (prime source of phycocyanin), are the examples of microalgae that have finally reached commercial-scale success [9,87,112]. Hundreds of many strains have been described in the literature as sources of phycobiliproteins. However, the lack of strain robustness or low productivity under outdoor environments has been typically cited as the cause of the failure of these strains in achieving commercial-scale production [25]. As a result, only selected strains can survive and perform well across a wide variety of culture conditions, including resistance to unfavorable short-term conditions, which can be cultivated outdoors [5]. Further additional research is needed to optimize the appropriate algal candidates to grow on a large scale and improve the productivity of valuable biomolecules.

### 3.7. Challenges and Approaches in the Phycobiliprotein Field

**Figure 12:**
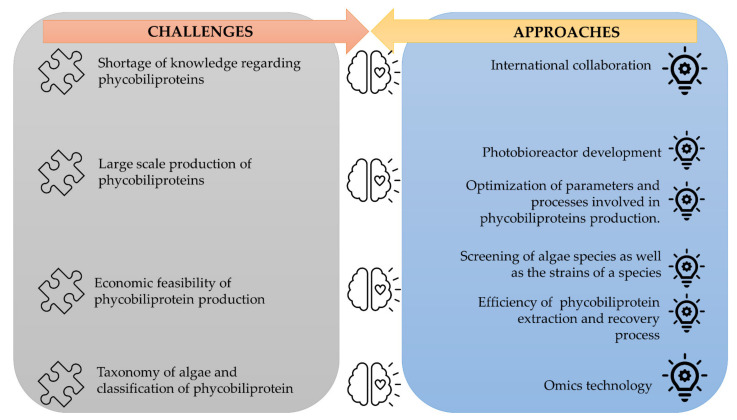
The challenges of phycobiliproteins research and its approaches.

The corpus of phycobiliprotein studies has been steadily enhanced and deepened due to the passion and efforts of researchers in studying phycobiliproteins. It gradually evolved from a fundamental and unitary topic to a multiperspective and sustainable development study field involving biology, chemistry, technology, and the environment. The market of phycobiliproteins will most likely continue to develop due to the rising natural product demand, the discovery of novel phycobiliproteins, advancements in the upstream and downstream processes, and expanding of the market potential [5,62]. The present study postulates that phycobiliprotein research would continue to be active and expand in bioactivity properties and applications. To meet the demand of the market, several strategies should be adopted (Figure 12). First, worldwide collaboration should be prioritized in order to conduct higher quality research. Second, most of the phycobiliproteins research is performed on a laboratory scale currently. Until now, China has achieved 200 tons/year production capacity, but the production is only limited to phycocyanin (https://www.binmei-global.com/about-us/ Assessed date: 28 June 2021). To obtain higher efficiency of growth and desired biomolecules, it is crucial to optimize relevant parameters in large-scale practical applications and develop optimal conditions of the photobioreactor [113,114]. Third, the major drawback in the use of phycobiliproteins is the high production cost. Additional screening of indigenous and novel cyanobacterial species/strains for high content of phycobiliproteins and fast-growing capability, effective harvesting, and economical purification methods may lower the production cost of phycobiliprotein. Another important aspect of phycobiliproteins research that needs to be highlighted is the taxonomy of algae species and the classification of phycobiliproteins. The use of an “omics” (genomics, metabolomics, proteomics, and transcriptomics) approach in research can enable the identification of species, detection of nutritional strategies, the interaction of symbiotic relationships with bacteria, and the biosynthesis of biomolecules, especially secondary metabolites [24]. These subject matters can help understand the metabolic pathways and regulatory mechanisms triggering the production of phycobiliproteins [113]. Besides, the development and utilization of robust platforms (Dictionary of Natural Products, MassBank, Global Natural Products Social Molecular Networking, and AntiBase) for data exchange and knowledge transfer/dissemination will undoubtedly facilitate new scientific knowledge and know-how [24].

## 4. Conclusions

The characteristics of phycobiliprotein-related publications from 1909 to 2020 and research hotspots were analyzed in this study. According to the findings, phycobiliproteins sparked the widespread interest of researchers. The drastic increase of phycobiliprotein research in terms of quantity over the last ten decades revealed a rapid growth, especially from 2017 onwards. This is consistent with the growth of the phycobiliprotein market. There were 2214 sources that published a total of 8296 publications. The majority of the publications were articles, and a few reviews were interspersed among them. Nearly all of the publications were written in English. The most subject area and journals that participated in the phycobiliproteins study were “biochemistry, genetics, and molecular biology” and “Journal of Applied Phycology”, respectively. The U.S.A. and China were the major contributors in the study of phycobiliproteins, while the U.S.A. and Germany were strongly interwoven in a worldwide research network. Glazer, Alexander N. from the University of California, U.S.A. was the top prolific author in phycobiliprotein research. Overall, the worldwide collaboration network has benefited from the rapid growth of phycobiliprotein research. According to keyword analysis trends, the scope of phycobiliprotein research includes the optimization of algae culture techniques and phycobiliprotein extraction processes, categorization of phycobiliproteins, phycobiliprotein biosynthesis pathway, phycobiliprotein bioactivities, and phycobiliprotein applications. The most commonly utilized model organisms in the phycobiliprotein study were *Arthrospira and Synechococcus*. The study trend using keyword burst detection revealed a growing concern regarding the extraction of phycobiliproteins and their bioactivities, especially their antioxidant properties. This is consistent with the keen interest in phycobiliproteins as bioactive compounds. Overall, this study provided essential information for researchers in seeking suitable institutions, initiating institutional research, or establishing collaborations in phycobiliprotein research. The findings might also aid researchers in identifying the trends and resources to construct impactful investigations that broaden the scientific frontier.

## Figures and Tables

**Table 1:** Main information of the retrieved phycobiliproteins data.

Description	Results
Period	1909–2020
Sources (journals, books, etc.)	2214
Documents	8296
Average years from publication	16.7
Average citations per documents	30.88
Average citations per year per doc	1.85
Annual growth rate of scientific production	9%
Authors	22,871
Authors of single-authored documents	331
Authors of multi-authored documents	22,540

**Table 2:** The top 10 prolific subject categories in the study of phycobiliprotein from 1909–2020.

Subject Area	Percentage (%)	Rank
Biochemistry, Genetics, and Molecular Biology	26.00	1
Agricultural and Biological Sciences	14.60	2
Medicine	12.60	3
Immunology and Microbiology	9.10	4
Chemistry	7.00	5
Environmental Science	5.90	6
Chemical Engineering	4.00	7
Engineering	3.32	8
Pharmacology, Toxicology, and Pharmaceutics	3.30	9
Physics and Astronomy	2.80	10

**Table 3:** The top 10 productive sources in the study of phycobiliprotein from 1909–2020.

Sources	TP	TP (%)	Cumulative (%)	TC	IF 2020	Rank by JIF	*h*- Index	Publisher
Journal of Applied Phycology	184	2.22	2.22	4122	3.215	Q1	93	Springer
Journal of Biological Chemistry	137	1.65	3.87	6672	5.157	Q1	477	American Society for Biochemistry and Molecular Biology
Journal of Phycology	127	1.53	5.40	5584	2.923	Q1	114	Wiley
Cytometry	121	1.46	6.86	5396	2.698	n/a	61	Wiley
Photochemistry and Photobiology	96	1.16	8.02	1979	3.421	Q3	120	Wiley
Photosynthesis Research	95	1.15	9.17	2173	3.573	Q1	99	Springer
Proceedings of the National Academy of Sciences of the United States of America	83	1.00	10.17	5923	11.205	Q1	699	United States National Academy of Sciences (U.S.A.)
Biochimica et Biophysica Acta—Bioenergetics	82	0.99	11.16	2556	3.991	Q2	154	Elsevier
Archives of Microbiology	81	0.98	12.14	3654	2.552	Q3	94	Springer
Biochemistry	77	0.93	13.07	3144	3.162	Q3	253	American Chemical Society

TP: total publications; TC: total citations; IF: impact factor; JIF: journal impact factor; n/a: not available.

**Table 4:** The characteristics of periodical publications of phycobiliprotein from 1909–2020.

PY	TP	TA	TA/TP	TR	TR/TP	TC	TC/TP
≤1960	33	46	1.39	11	0.03	841	25.48
1961–1970	133	161	1.21	413	3.11	3736	28.09
1971–1980	337	422	1.25	5691	16.89	11,462	34.01
1981–1990	821	1547	1.88	15,380	18.73	30,248	36.84
1991–2000	1553	4175	2.69	35,553	22.89	70,619	45.47
2001–2010	1909	6410	3.36	58,387	30.59	81,576	42.73
2011–2020	3510	14,000	3.99	158,176	45.06	57,738	16.45

PY: year of publication; TP: total publications; TA: total authors; TA/TP: authors per publication; TR: total references; TR/TP: average references per publication; TC: total citations (until 2020); TC/TP: average citations per publication.

**Table 5:** The top 10 most cited publications related to phycobiliproteins from 1909–2020.

Title of Publications	Journal	TC	PD	References
The Chemistry behind Antioxidant Capacity Assays	Journal of Agricultural and Food Chemistry	4010	Feb 2005	[30]
Commercial Applications of Microalgae	Journal of Bioscience and Bioengineering	2574	Oct 2006	[31]
Antioxidant and Prooxidant Behavior of Flavonoids: Structure-Activity Relationships	Free Radical Biology and Medicine	2038	June 1997	[32]
Development and Validation of an Improved Oxygen Radical Absorbance Capacity Assay Using Fluorescein as the Fluorescent Probe	Journal of Agricultural and Food Chemistry	2024	Jan 2001	[35]
HLA-E Binds to Natural Killer Cell Receptors CD94/NKG2A, B and C	Nature	1592	Feb 1998	[36]
Determination of Lymphocyte Division by Flow Cytometry	Journal of Immunological Method	1439	Feb 1994	[37]
Resolution and Characterization of Pro-B and Pre-Pro-B Cell Stages in Normal Mouse Bone Marrow	Journal of Experimental Medicine	1317	May 1991	[38]
Accessing Genetic Information with High-Density DNA Arrays	Science	1309	Oct 1996	[39]
Oxygen-Radical Absorbance Capacity Assay for Antioxidants	Free Radical Biology and Medicine	1256	Jan 1993	[40]
A New Approach to Protein Fold Recognition	Nature	1035	July 1992	[34]

TC: total citation; PD: publication date.

**Table 6:** The top 10 productive countries in phycobiliproteins research from 1909–2020.

Country	TP (%)	TC	TC/TP	*h*	Number of Publications						
					≤1960	1961–1970	1971–1980	1981–1990	1991–2000	2001–2010	2011–2020
United States of America (U.S.A.)	2240 (27.00)	103,660	46.28	132	11	65	138	353	538	470	665
China	1177 (14.19)	17,961	15.26	60	0	0	0	8	89	289	791
Germany	775 (9.34)	24,613	31.76	71	0	9	46	112	179	201	228
India	637 (7.67)	13,752	21.59	56	0	1	7	21	79	148	381
Japan	534 (6.44)	13,814	25.87	59	6	7	23	61	116	151	170
France	441 (5.32)	20,542	46.58	71	0	3	12	41	111	130	144
United Kingdom	382 (4.60)	17,008	44.52	64	3	10	7	46	100	91	125
Spain	299 (3.60)	9303	31.11	52	0	0	0	10	61	86	142
Italy	264 (3.18)	8229	31.17	47	0	1	4	8	57	59	135
Canada	246 (2.97)	9106	37.02	54	0	1	4	25	62	59	95

TP: total publications; TC: total citations; TC/TP: average citations per publication; *h*: h-index.

**Table 7:** The top 10 productive institutions in phycobiliproteins research from 1909–2020.

Institution	Country	TP (%)	TC	TC/TP	*h*	Number of Publications						
						≤1960	1961–1970	1971–1980	1981–1990	1991–2000	2001–2010	2011–2020
Chinese Academy of Sciences	China	352 (4.24)	6599	18.75	44	0	0	0	6	63	96	187
CNRS (Centre National de la Recherche Scientifique)	France	150 (1.80)	6658	44.39	45	0	3	5	14	34	36	58
Ludwig-Maximilians-Universität München	Germany	132 (1.59)	3703	28.05	35	0	1	20	36	30	24	21
University of California, Berkeley	U.S.A.	130 (1.57)	7574	58.26	49	1	2	13	52	39	6	17
Russian Academy of Sciences	Russia	105 (1.27)	2080	19.81	22	0	3	10	6	22	21	43
Ministry of Education China	China	94 (1.13)	1089	11.59	18	0	0	0	0	0	10	84
The University of Tokyo	Japan	91 (1.10)	2981	32.76	29	2	5	11	10	14	25	24
University of Chinese Academy of Sciences	China	91 (1.10)	1571	17.26	24	0	0	0	0	0	39	52
New York State Department of Health	U.S.A.	81 (0.98)	2141	26.43	22	0	10	25	22	20	3	1
Lomonosov Moscow State University	Russia	73 (0.88)	1078	14.77	19	0	0	2	4	17	16	34

TP: total publications; TC: total citations; TC/TP: average citations per publication; *h*: h-index.

**Table 8:** The top 10 productive authors in phycobiliproteins research from 1909–2020.

Author	Country	Institutions	TP (%)	*h*	Email	Number of Publications						
						≤1960	1961–1970	1971–1980	1981–1990	1991–2000	2001–2010	2011–2020
Glazer, Alexander N.	U.S.A.	University of California	118 (1.42)	65	glazer@berkeley.edu	0	0	28	57	28	5	0
Scheer, Hugo	Germany	Ludwig-Maximilians-Universität München	89 (1.07)	49	hugo.scheer@lmu.de	0	0	6	23	26	16	18
Bryant, Donald A.	U.S.A.	Pennsylvania State University	64 (0.77)	72	dab14@psu.edu	0	0	5	16	14	12	17
Qin, Song	China	Yantai Institute of Coastal Zone Research, Chinese Academy of Sciences,	62 (0.75)	35	sqin@yic.ac.cn	0	0	0	0	1	25	36
Berns, Donald S.	Israel	Israel Ministry of Health	59 (0.71)	24	d.berns@netvision.net.il	0	14	28	13	4	0	0
Zhao, Kaihong	China	Huazhong Agricultural University	55 (0.66)	26	kaihongzhao@mail.hzau.edu.cn	0	0	0	0	5	20	30
MacColl, Robert	U.S.A.	Wadsworth Center for Laboratories and Research	52 (0.63)	25	maccoll@wadsworth.ph.albany.edu	0	0	14	15	19	3	1
Madamwar, Datta B.	India	Sardar Patel University	45 (0.54)	49	datta_madamwar@yahoo.com	0	0	0	0	1	8	36
Bekasova, Olga D.	Russia	Bach Institute of Biochemistry,	40 (0.48)	7	bekasova@inbi.ras.ru	0	3	14	8	1	6	6
Gantt, Elisabeth	U.S.A.	University of Maryland	40 (0.48)	43	egantt@umd.edu	0	2	19	15	3	0	1

TP: total publications; *h*: h-index.

**Table 9:** The clusters and themes derived from keywork co-occurrence network in the phycobiliproteins research.

Clusters	Co-Words	Themes
Cluster I (21 items)	Algae, *Arthrospira platensis*, biomass production, bioreactor, biotechnology, culture media, extraction, isolation and purification, light intensity, light quality, optimization, *oscillatoria*, pH, photobioreactor, phycobiliprotein, *Porphyridium, Porphyridium cruentum,* process optimization, salinity, spirulina platensis and temperature	Optimization of cyanobacteria cultivation and phycobiliprotein harvesting process
Cluster II (12 items)	Allophycocyanin*, Anabaena* sp.*, Anabaena variabilis,* cyanobacteria*,* cyanobacterium*, Fremyella diplosiphon*, *Mastigocladus laminosu, nostoc* sp., phycocyanin, phycoerythrocyanin, *Synechococcus elongatus* and *Synechocystis* sp.	Classification of phycobiliproteins from different cyanobacteria
Cluster III (11 items)	Biliverdin, biliverdine, biosynthesis, ferredoxin, *fremyella diplosiphon*, gene expression, phycobilins, phycocyanobilin, phycoerythrin, phycoerythrobilin and *prochlorococcus*	Gene expression of the phycobiliprotein biosynthesis pathway
Cluster IV (4 items)	Anti-inflammatory activity, antineoplastic agent, antioxidant, antioxidant activity	Bioactivities of phycobiliprotein
Cluster V (4 items)	Biofuels, fluorescent dye, fluorescent dyes, fluorescent spectroscopy	Applications of phycobiliprotein

## Data Availability

Not applicable.
